# Introducing a novel intraoral mandibular nerve block technique for loco-regional analgesia in camels (*Camelus dromedarius*): a cadaveric study using computed tomography

**DOI:** 10.1186/s12917-024-03885-4

**Published:** 2024-02-03

**Authors:** Mohamed Marzok, Mohamed A. Nazih, Adel I. Almubarak, Zakriya Al Mohamad, Ibrahim A. Emam, Mohamed W. El-Sherif

**Affiliations:** 1 Department of Clinical Sciences, College of Veterinary Medicine, King Faisal University, Al-Ahsa, Saudi Arabia; 2 Department of Surgery, Faculty of Veterinary Medicine, Kafrelsheikh University, Kafrelsheikh, Egypt; 3 Department of Anatomy, Faculty of Veterinary Medicine, New Valley University, Elkharga, New Valley 72511 Egypt; 4 Department of Surgery, Anesthesiology and Radiology, Faculty of Veterinary Medicine, Cairo University, Giza, Egypt; 5 Department of Surgery, Faculty of Veterinary Medicine, New Valley University, Elkharga, New Valley 72511 Egypt

**Keywords:** Analgesia, Computed tomography, Camel, Mandibular, Nerve block

## Abstract

The aim of this study was to introduce a novel intraoral technique for performing mandibular nerve blocks in dromedary camels (*Camelus dromedarius*). In this study, 18 adult camel skulls of varying ages and breeds were examined to determine the position of the mandibular foramen. Using a Vernier caliper, three dimensions in millimeters were measured: (1) the distance between the mandibular foramen (MF) and the caudal edge of the third molar tooth at the occlusal surface level, (2) the distance between the MF and the rostral edge border of the mandible’s ramus (RER) at the occlusal surface level, and (3) the distance between the MF and the ventral margin border of the mandible (VM). The technique was evaluated using five intact camel cadaver heads (*n* = 5), and a total of ten mandibular nerve blocks were described. An 18-gauge 80-mm Tuohy needle was inserted into the mouth commissure and advanced caudally while injecting a saline-methylene blue solution. The accuracy of the injection was confirmed through the infiltration of the contrast dye into the target area using computed tomography (CT) and post procedural dissection. Anatomical study of the mandibular nerve site was performed to aid the blind insertion of the needle. The findings contribute to the development of veterinary anesthesia techniques and provide anatomical considerations for clinicians performing oral surgeries in sedated camels. The results demonstrated the successful implementation of the intraoral technique, highlighting its efficacy and reliability in achieving local anesthesia for oral surgeries involving the lower jaw and teeth in sedated camels. Further research studies are needed to evaluate the long-term efficacy and safety of the technique and to compare it with existing approaches.

## Background

The mandibular nerve block is widely employed to achieve anesthesia in the lower jaw [1, 2]. The mandibular nerve, also known as the inferior alveolar nerve, is a branch of the trigeminal nerve that provides sensory innervation to the lower teeth, gingiva, and mandible in camels [3]. Injection of local anesthetics around the mandibular nerve can temporarily block sensation to these areas, allowing for painless dental, lips and mandibular surgeries [4]. This block is particularly valuable for different mandibular diagnostic and surgical procedures in camels [5–10].

The traditional method to block the mandibular nerve in camel is to use an extra-oral approach in which the nerve is desensitized at mandibular foramen which is situated on the medial aspect of the mandible where two imaginary lines cross each other (first straight line is drawn from the point of union of two parietal crests vertically downwards and second line is drawn from the masticatory surface lower check teeth). An eight cm (8 cm) long and 16-gauge needle is introduced blindly at the medial aspect of the ventral margin of the mandible. The needle is advanced along the medial surface of the vertical ramus of the mandible to the depth and in the direction of the point of intersection of the imaginary lines. Thereafter 15 to 20 ml of 2% lidocaine HCL is injected [11, 12].

Challenges with the extra-oral technique include the difficulty in precise anesthetic deposition near the mandibular foramen, with the risk of needle movement. Thorough anatomical knowledge is crucial to prevent inadvertent damage to surrounding structures, including blood vessels [4, 11]. .

The intraoral technique for mandibular nerve block has been used increasingly on a variety of various species, including humans, equine and cattle [13–15]. However, there is limited knowledge regarding the safety and accuracy of mandibular nerve blocks in camels. Computed Tomography (CT) denotes a computerized x-ray imaging technique aimed at producing cross-sectional images, or “slices,” providing clinicians with more intricate information compared to conventional x-rays. The machine’s computer gathers successive slices, which can then be digitally compiled to create a three-dimensional (3D) image of the patient, facilitating the enhanced identification of fundamental anatomical structures and precise details of the procedures [16]. The aim of this study is to estimate the average measurements of the location of the mandibular foramen on camel skulls and to introduce a novel intraoral technique for performing mandibular nerve blocks in camels using (CT).

## Materials and methods

### Anatomical determination of the mandibular foramen location

To determine the position of the mandibular foramen, 18 adult camel skulls of various ages and breeds were studied. The dentition was used to estimate ages according to Belloa et al., [17]. All measurements were performed on the right side. A Vernier caliper was used to measure three dimensions in millimeters: (1) the distance between the mandibular foramen (MF) and the caudal edge of the third molar tooth at the occlusal surface level. (2) the distance between the MF and the rostral border of the mandible’s ramus (RER) at the occlusal surface level. (3) the distance between the MF and the ventral border of the mandible (VM).

### Specimen acquisition and experimental procedure

Five intact camel cadaver heads were obtained from a local abattoir for use in this study. A total of ten mandibular nerve blocks were performed as part of the experimental procedure. All heads were prepared by extensively opening the mouth and securing it in an open position using a mouth gauge. To facilitate the nerve block, the tongue was firmly grasped on the contralateral side of the targeted nerve. The procedure was conducted under appropriate illumination conditions.

### Intraoral mandibular nerve block technique

**Fig. 1:**
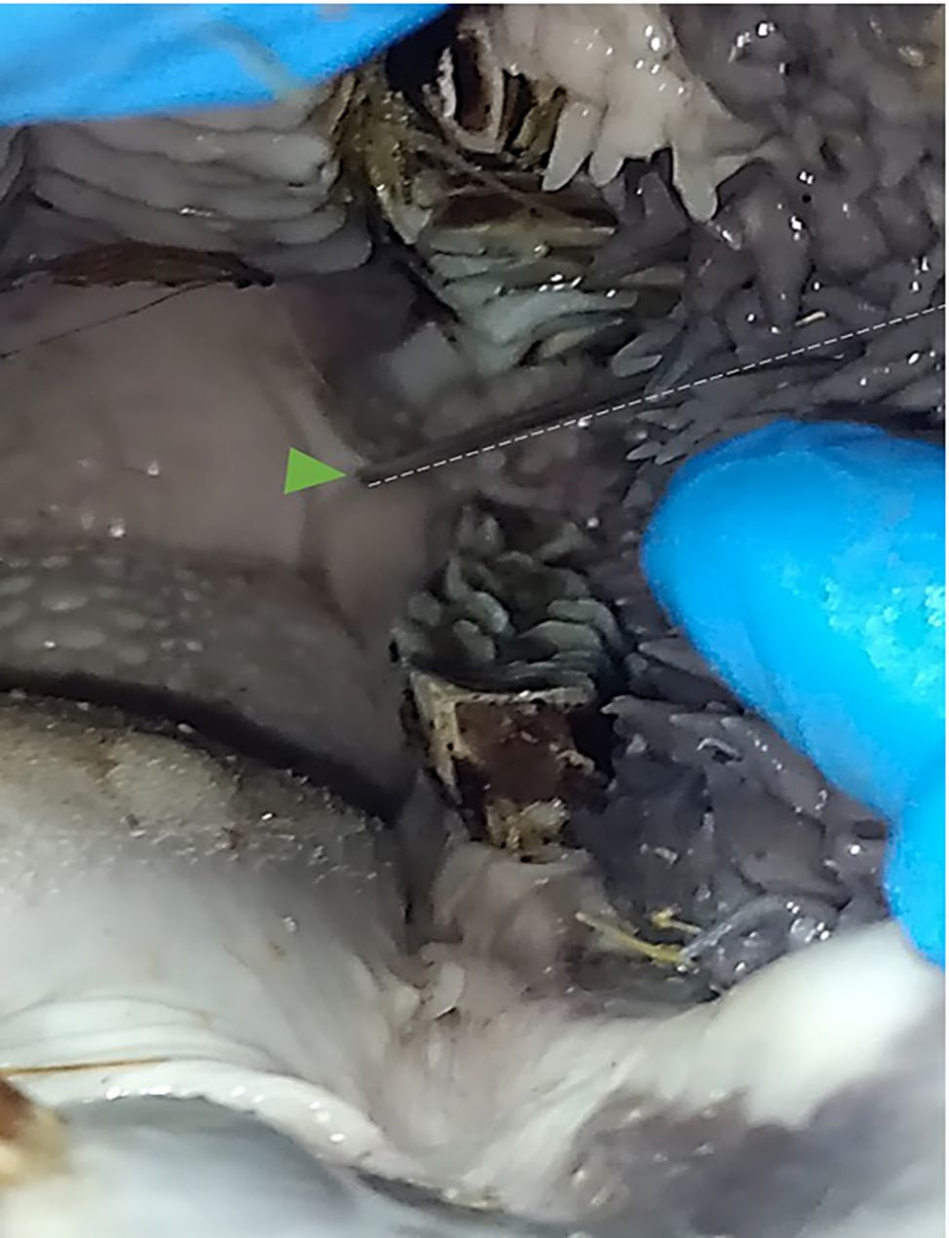
Intraoral image shows the point of needle insertion slightly above occlusal surface of the mandibular third molar tooth (just lateral to Pterygomandibular fold) (green arrow head)

In this procedure, a specialized 18-gauge × 80 mm Tuohy spinal needle, with an elastic tube for external injection, was employed. The needle was securely held from the hub and inserted into the pterygomandibular fold. Carefully, it gradually advanced in a caudal direction and slightly above occlusal surface of the mandibular third molar tooth on the medial aspect of the vertical mandibular ramus. Once the needle reached its designated position, a meticulous injection of methylene blue-saline solution was administered (Fig. 1).

### Computed tomography study

After the spinal needle was inserted, all heads were positioned on the scanner table on its rostral aspect towards the gantry. They were then scanned using computed tomography (CT) 16 slice scanner (CT-W450-10 A, Hitachi Medical Corporation, Japan) 120KV, 100–350 mAs to determine the accuracy of the blind positioning of the needle nearby the mandibular foremen. A solution containing 6 ml of contrast medium (saline-methylene blue 3:1) was injected while simultaneously withdrawing the needle. The contrast medium was administered through extension tubing attached to the spinal needle prior to mucous membrane penetration to prevent or decrease the risk of needle movement during syringe attachment.

### Data collection and analysis

Following the injection procedure and CT scanning, an anatomical dissection was conducted to delineate the path of the methylene blue-dyed saline solution utilized for staining the anesthetized area. The infiltrated area was carefully examined and measured to assess the extent of anesthesia achieved. Descriptive data were expressed as mean + SEM and statistically analyzed using SPSS (Version 28.00, IBM, USA).

## Results

### Surgical anatomy (documentation the location and dimensions of MF)

**Fig. 2:**
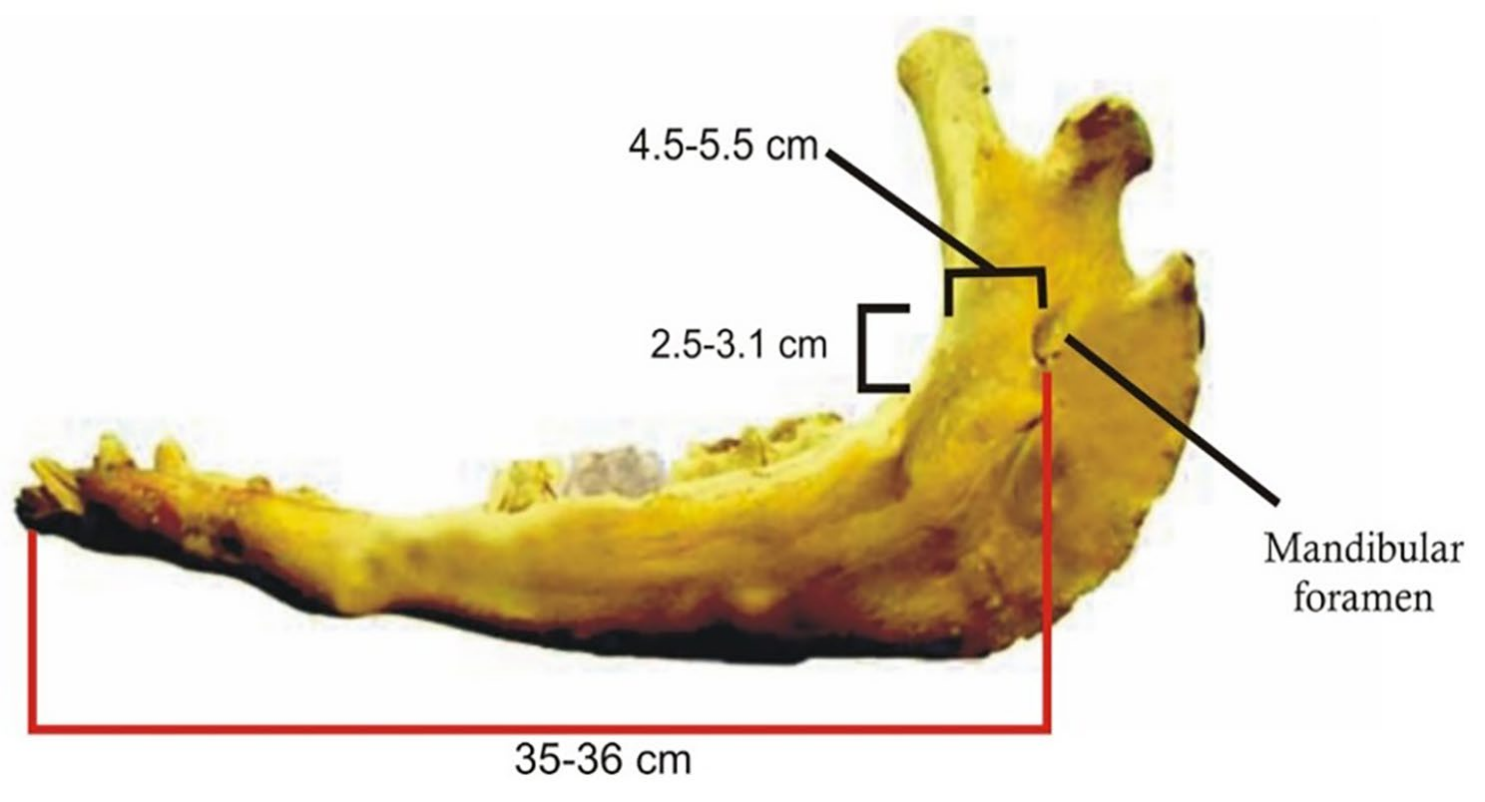
A photograph showing dimensional measurements of the mandibular foramen (Medial view)

Following anatomical dissection of the heads and based on the observation of rostral dentition, the mean estimated age was determined to be 9 ± 4.3 years. The mandibular foramen, situated on the medial aspect of the vertical ramus of the mandible (Fig. 2), is anatomically located at the midpoint between its rostral and caudal borders. The width of the foramen is approximately 10 ± 2 mm, and it is positioned approximately 45 ± 10 mm from the rostral border of the mandibular ramus. Extending towards the caudal border, it reaches approximately 50 ± 4 mm. Furthermore, the mandibular foramen is found dorsally to the level of the occlusal surface of the last molar tooth by approximately 25 ± 6 mm.

**Fig. 3:**
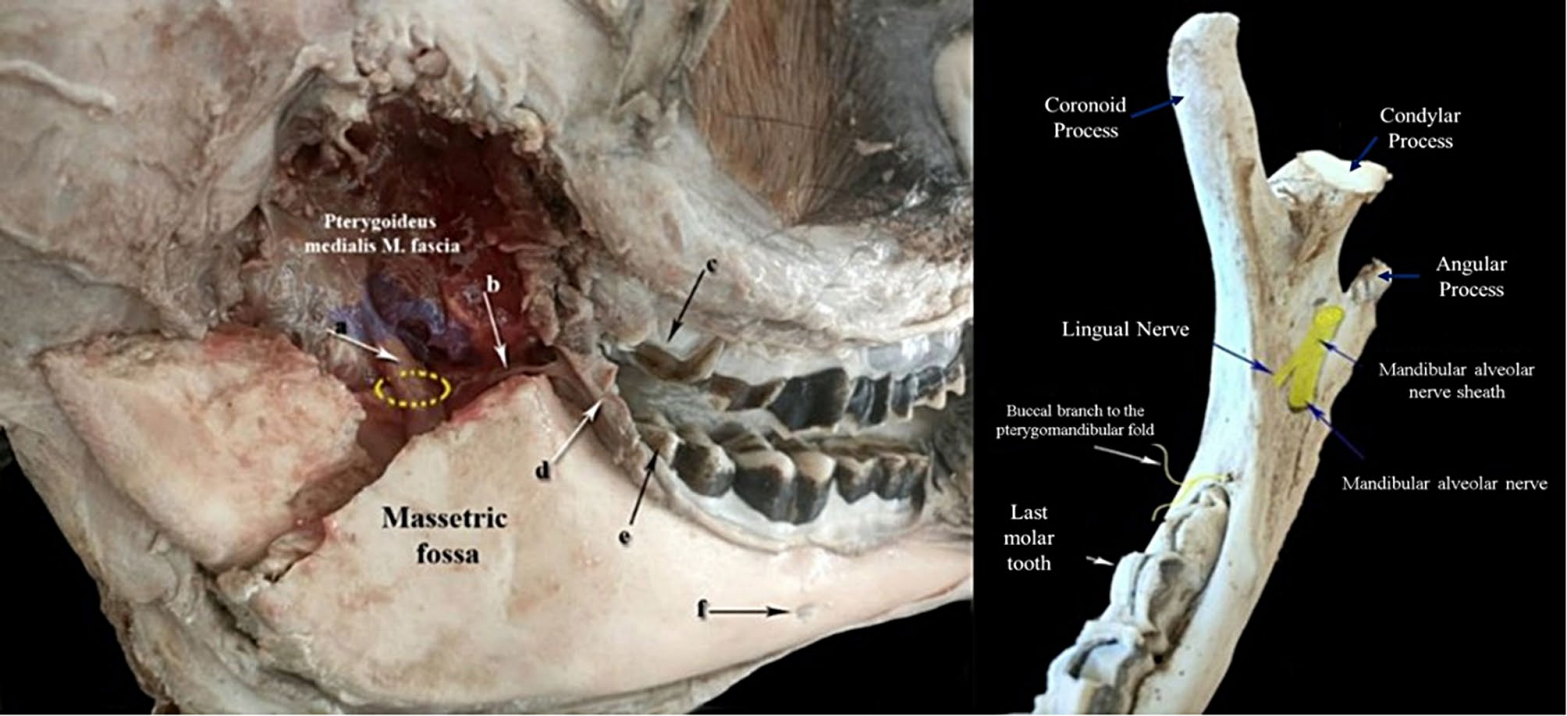
The image to the left shows deep dissection of the lateral aspect of the camel face showing (**a**) Mandibular alveolar nerve common sheath, (**b**) Buccal branch to the pterygomandibular fold, (**c**) the last upper molar tooth, (**d**) Pterygomandibular fold, (**e**) The last lower molar tooth, and (**f**) caudal mental foramen. The dotted yellow circle indicates the mandibular foramen, and the colored area indicates the injected material. The image to the right shows the ramus of the mandible of adult camel (dorso-medial view)

The anatomical dissection showed that, on the medial aspect of the ramus of the mandible (pterygoideus fossa), the common mandibular alveolar nerve sheath exists. The latter comprises the mandibular alveolar and lingual nerves of trigeminal. The sheath pierces in the ventrolateral direction the pterygoideus medialis muscle fascia, where it detaches the lingual nerve which traverses in rostro- ventrolateral direction, caudally to the pterygomandibular fold by about 40–45 mm. The mandibular alveolar nerve descends in the mandibular canal through the mandibular foramen, where it sends fine branch to the pterygomandibular fold. The branch passes rostrally to emerge from the rostral border of the vertical ramus to reach the buccal mucosa (Fig. 3).

**Fig. 4:**
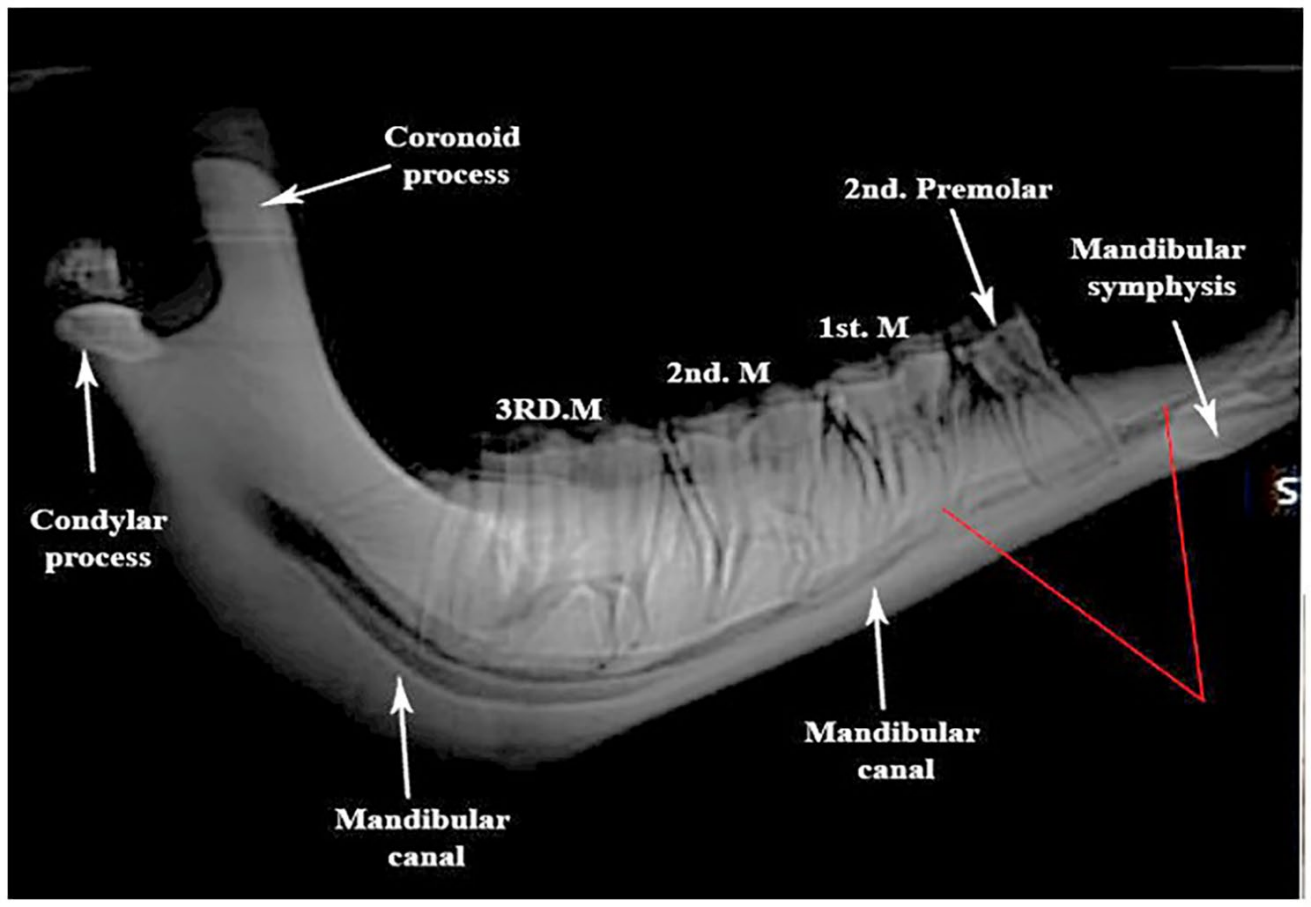
Computed tomography (CT scan) of the mandible, (lateral view)

Computed tomographic anatomical assessment reveals thickening of the mandibular wall, specifically marked thinning of the ventral wall of the horizontal mandibular ramus. The thinning is clearly visible in the area between the 1st and 2nd premolars, coinciding with the configuration of the widely configured mandibular canal at that level (Fig. 4).

**Fig. 5:**
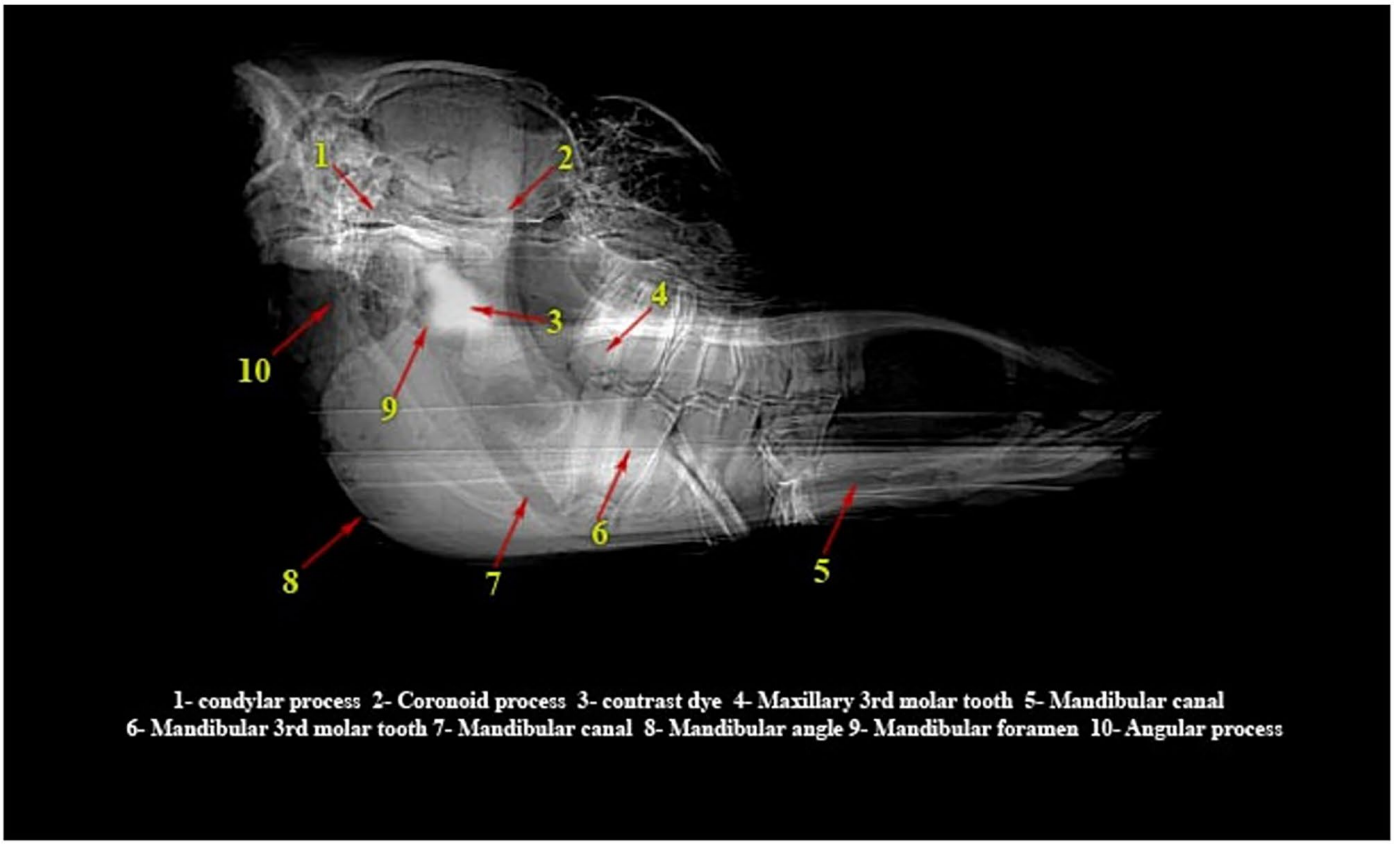
Computed tomography of the camel head showing successive deposition of contrast medium just before the mandibular foremen

Computed tomography confirmed initially the accurate positioning of the needle and secondly the precise infiltration of the dye on the targeted site of the mandibular foramen of all of five cases with mean area of dye infiltration are being 2 cm², with a standard error mean of 0.5 cm². The minimum and maximum sizes of the infiltrated areas were 1.8 cm² and 2.3 cm², respectively (Fig. 5).

The intraoral mandibular nerve block technique successfully described the mandibular nerve block in all five cases. CT confirmed the infiltration of the dye on the targeted site of the mandibular foramen with mean diameter of infiltration being 44 (+ 5) mm (range = 18 to 23 mm). Following CT assessment dissection of the injection site revealed constant infiltration of the mandibular nerve and foramen with methylene blue-saline.

## Discussion

Mandibular nerve block remains an important anesthesia technique for oral surgeries involving the lower jaw and teeth in animals [2, 18]. Correct anatomical knowledge and aseptic technique are essential for performing mandibular nerve blocks in camels. The mandibular foramen, where the mandibular nerve enters the mandible, is located on the medial surface of the ramus of the mandible in camels [19].

The mandibular nerve is desensitized at the mandibular foramen to induce analgesia during the treatment of the injuries related to the lower jaw and lower tooth. By avoiding desensitization, the lingual branch of the mandibular nerve and glossopharyngeal nerve, the tongue can be protected from accidental injury after surgery [20–22]. Access to this foramen is achieved by extraoral insertion of a needle through the ventral aspect of the mandible at the level of the last molar tooth as described by [22]. Correct placement of the needle is critical, as inaccurate injection can lead to lack of efficacy or injury to nearby structures like blood vessels [4]. Injecting the anesthesia solution in front of the mandibular nerve may decrease the likelihood of puncturing the inferior alveolar artery. By employing the technique described in this study, there is potential to mitigate the risk of post-procedural tongue paralysis and self-inflicted trauma through the judicious use of a smaller quantity of anesthetic solution and precise placement. This perceived advantage of the intraoral technique over the extraoral method warrants further substantiation through additional clinical research to validate the hypothesis.

The present study focused on the introduction of a novel intraoral technique for performing mandibular nerve blocks in camels and provided an in-depth examination of the surgical anatomy, technique, and results. The successful implementation of the intraoral mandibular nerve block technique in all five cadavers, employing dye in a simulated study, indicates the feasibility and reliability of the technique. However, the transition to clinical application requires careful consideration of challenges, including the potential risk of biting, the relatively long camel head, navigating between maxillary and mandibular cheek teeth, and the short length of the needle. The confirmation of accurate injection through the infiltration of the methylene blue-dyed saline solution further validates the precision of the technique. These results are significant as they demonstrate the potential for utilizing this novel technique in mandibular surgeries involving the lower jaw and teeth in camels.

The anatomical assessment revealed information regarding the location and dimensions of the mandibular foramen in camels. The consistent position of the foramen on the medial aspect of the vertical ramus of the mandible, coupled with its specific measurements, provides crucial guidance for the accurate identification and targeting of the nerve during the intraoral nerve block. This anatomical knowledge is fundamental for the successful application of the technique and emphasizes the importance of understanding the specific anatomy of the mandibular region in camels.

In the present study, the distance from the caudal border of the mandible to the level of the MF and from the latter to rostral border of mandibular ramus was 5 ± 0.4 cm and 4.5 ± 1 cm. In addition, the MF was found dorsally to the level of the last molar tooth by approximately 2.5 ± 0.6 cm. Similarly, in concordance with previous findings in dromedary camels, comparable measurements were observed. The distance from the caudal border of mandible to the level of mandibular foramen, and from the latter to the border of mandibular angle was about 3.7 and 4.3 cm [23–25]. From the clinical point of view, these data are considered important landmarks for achieving accurate regional analgesia of the mandibular nerve. This will ultimately lead to desensitization of the lower lip and all the lower jaw with its teeth on the blocked side.

There is a potential issue with piercing the nerve when using the needle in both the extra-oral and intraoral techniques, which may result in a sudden upward movement of the head. Touhy needles were used in the present study, as they are less prone to accidentally cutting or piercing the nerve during injection.

The computed tomographic assessment further contributed to the understanding of the mandibular anatomy by highlighting the thickening of the mandibular wall and the thinning of the ventral wall of the horizontal mandibular ramus. This observation, particularly between the 1st and 2nd premolars, correlates with the configuration of the mandibular canal, which holds clinical significance.

The CT imaging of cadaver heads demonstrated that the intraoral approach to the IANB allowed a precise placement of the Methylene blue-saline at the entrance of the mandibular foramen. The needle placement at the mandibular foramen was easily recognized and the distribution of the contrast material adequately infiltrated the area of the mandibular nerve. The injected volume utilized in this study, at 6 ml, is notably lower than the recommended amount for the extraoral approach (15–20 ml) [11, 12], yet aligns with volumes reported in live buffalo [23]. However, additional clinical investigations are imperative to ascertain the efficacy of this specific anesthetic volume in camels.

The descriptive analysis provides additional information about the size of the infiltrated area and dimensions of the mandibular foramen, which are relevant for surgical planning and management. The anatomical and computed tomographic assessments further confirm the anatomical location of the mandibular foramen and provide insights into the thickness of the mandibular wall, which may have implications for the success and safety of the nerve block technique. The mean size of the infiltrated area was 2 cm², indicating a reasonably consistent spread of local anesthesia.

The current novel intraoral technique in adherence with the existing techniques described in equine and cattle [14, 26] seems to offer potential advantages. The intraoral approach provides direct access to the mandibular foramen, facilitating accurate needle placement and reducing the risk of injury to nearby blood vessels. Additionally, the technique eliminates the need for extra-oral insertion of a needle, potentially improving patient comfort and minimizing the risk of contamination. However, further studies comparing the efficacy and safety of the intraoral technique to other approaches are warranted to establish its superiority.

It is important to acknowledge certain limitations of this study. The sample size was relatively small, involving only five cadavers. A larger sample size would provide more robust evidence of its efficacy. Additionally, the study focused on the anatomical aspects and initial outcomes of the technique. Long-term follow-up studies and comparison with other techniques on alive animals are needed to evaluate the duration and efficacy of anesthesia, postoperative complications, and patient recovery.

## Conclusions

In conclusion, this study successfully introduced a novel intraoral technique for performing mandibular nerve blocks in camels. The findings of this study contribute to the advancement of veterinary anesthesia techniques and provide valuable insights for clinicians performing oral surgeries in camels. However, it is necessary to conduct controlled clinical trials to evaluate the feasibility and efficacy of implementing this approach in real-world clinical practice.

## Data Availability

All data collected or analyzed during this study are included in this published paper.
